# Identification of nitric oxide (NO)-responsive genes under hypoxia in tomato (*Solanum lycopersicum* L.) root

**DOI:** 10.1038/s41598-020-73613-z

**Published:** 2020-10-05

**Authors:** Vajiheh Safavi-Rizi, Marco Herde, Christine Stöhr

**Affiliations:** 1 Department of Plant Physiology, Institute of Botany and Landscape Ecology, University of Greifswald, Greifswald, Soldmannstrasse 15, 17487 Greifswald, Germany; 2 Department of Molecular Nutrition and Biochemistry of Plants, Institute of Plant Nutrition, Leibniz University Hannover, Herrenhäuser Strasse 2, 30419 Hannover, Germany

**Keywords:** Plant sciences, Plant signalling, Plant stress responses

## Abstract

Flooding periods, as one probable consequence of climate change, will lead more frequently to plant hypoxic stress. Hypoxia sensing and signaling in the root, as the first organ encountering low oxygen, is therefore crucial for plant survival under flooding. Nitric oxide has been shown to be one of the main players involved in hypoxia signaling through the regulation of ERFVII transcription factors stability. Using SNP as NO donor, we investigated the NO-responsive genes, which showed a significant response to hypoxia. We identified 395 genes being differentially regulated under both hypoxia and SNP-treatment. Among them, 251 genes showed up- or down-regulation under both conditions which were used for further biological analysis. Functional classification of these genes showed that they belong to different biological categories such as primary carbon and nitrogen metabolism (e.g. glycolysis, fermentation, protein and amino acid metabolism), nutrient and metabolites transport, redox homeostasis, hormone metabolism, regulation of transcription as well as response to biotic and abiotic stresses. Our data shed light on the NO-mediated gene expression modulation under hypoxia and provides potential targets playing a role in hypoxia tolerance. These genes are interesting candidates for further investigating their role in hypoxia signaling and survival.

## Introduction

Nitric oxide (NO) is an important signaling molecule involved in a wide range of physiological processes during plant development. NO has been reported to play a role in photomorphogenesis and flowering^1^, development of lateral root^2^, organ growth^3^ and senescence^4^. Moreover, NO has been shown to be involved in response to different abiotic and biotic stresses such as heat^5^, drought^6–8^, salinity^9,10^, ozon^11^, heavy metal^12,13^, pathogen attack^14^ as well as flooding and hypoxia^15–17^.

NO is a highly reactive molecule and therefore its conversion to a non-toxic molecule is crucial to avoid cell toxicity^18^. NO level in plant cell is mainly regulated via *S*-nitrosoglutathione reductase (GSNOR) activity^19^. Interestingly, *S*-nitrosylation of GSNOR1 itself at Cys10, resulted in conformational changes and direct interaction with ATG8 leading to the autophagy of GSNOR1 in Arabidopsis^20,21^. Furthermore, phytoglobins (PGBs) have been reported to be involved in NO scavenging and its conversion to nitrate^22,23^.

*S*-Nitrosoglutathione (GSNO) is one of the important NO-bioactive molecules inside the cell involved in NO-mediated post-translational modifications (PTMs) such as cysteine *S*-nitrosylation, metal nitrosylation and tyrosine nitration of target proteins^18,24,25^. Moreover, NO can lead to fatty acid nitration and nitro fatty acid production, which might further contribute to plant NO signaling^26–28^.

NO can be synthesized via oxidative or reductive pathways, either enzymatic or nonenzymatic. NO synthase (NOS) is the enzyme involved in NO biosynthesis in mammals^29^. Although in higher plants, NO synthase inhibitors decrease the production of NO and citrulline from L-arginine, the NOS homologe in the plant cells has not yet been identified, except in *Ostreococcustauri*, a single cell alga^24,30^. In plants, Xanthine oxidoreductase (XOR), a key enzyme involved in purine catabolism, has been shown to convert nitrite to NO in vitro in the presence of reducing substrates NADH or xanthine in an oxygen dependent manner^31–33^. The cytosolic nitrate reductase (NR), the key enzyme involved in nitrate assimilation which converts nitrate to nitrite, may also catalyze the conversion of nitrite to NO^34^. Cytochrome c oxidase, located in the inner membrane of mitochondria, is involved in NO synthesis from nitrite^35,36^. Loss-of-function mutant of Arabidopsis NOA1 (*noa1*) showed reduced level of NO^37^. NOA1 is a plastid targeted GTPase, which is associated with ribosome function. Therefore, NO-deficient phenotype of *noa1* mutant is possibly an indirect effect due to the hindered chloroplast function^37^. Oxygen dependent enzymatic extracellular NO production has been suggested to be important for sensing the availability of nitrate. In this direction, a Nitrite-NO reductase (NiNOR) activity has been discovered in root plasma membrane (PM), which uses the nitrite provided by PM bound NR as substrate under low oxygen condition^16,38^. Recently, a new enzymatic NO producing route has been reported in *Chlamydomonas reinhardtii* which produces NO via activity of two molybdenum cofactor enzymes, NOFNiR (nitric oxide-forming nitrite reductase) and NR^39,40^, however, its role in NO production in higher plants is still not clear.

Conversion of nitrite to NO can also occur non-enzymatically under low pH in apoplast and plastids^37,41,42^. This complexity of plant NO production in different parts of the cell via various enzymatic and non-enzymatic reactions, complicates investigating the NO effect using mutant lines. This is even more problematic in crops compared to *Arabidopsis* due to their genome complexity. Hence, NO donors are used as an alternative tool for NO studies. Among different NO donors, sodium nitroprusside (SNP) is widely used to study the effect of NO. Beside its lower cost compared to other NO donors, SNP releases NO continuously and for longer period of the time, which might be of advantage for biological studies^43–45^.

The effect of NO during flooding stress has been intensively addressed in a recent review^46^. Former studies have shown that NO-mediated *S*-nitrosylation of several proteins such as ERFVIIs, phytoglobins, cytochrome *c* oxidase (COX), aconitase, and ascorbate peroxidase (APX1) might be associated with flooding signaling and tolerance^46–48^. Moreover, it has been reported that under hypoxia, NO plays a pivotal role by regulation of COX and alternative oxidase (AOX) activity and therefore, mitochondrial oxygen consumption^49^.

Endogenous NO level plays a role in regulation of transcription under hypoxia through the N-degron pathway^48,50^. It has been shown that degradation of MC-ERFVII transcription factors (TFs) occurs under NO availability via the N-degron pathway. Adversely, ERFVII TFs are stabilized in the absence of NO^48^.

Tomato is an economically important crop plant which is sensitive to flooding induced hypoxia^51,52^. However, our understanding of the precise mechanism of hypoxia tolerance and in particular the role of NO during this process is not complete. Moreover, our knowledge about NO effect on gene expression regulation in tomato during hypoxia is still scarce. Therefore, identification of NO-responsive genes under hypoxia is essential for improving flooding stress tolerance in tomato.

In our former study, we identified short-(6 h) and long-term (48 h) hypoxia-responsive genes in tomato (*cv.* Moneymaker) roots^53^. Those data suggest a distinct temporal transcriptional response. While short term hypoxia resulted in transcriptional acclimation, hypoxia progression resulted in a transcriptional reprogramming to support an escape mechanism probably through aerenchyma and adventitious root formation^53^. This indicates the ability of a cultivated crop such as tomato to temporally adjust its response mechanism to hypoxia, both metabolically and anatomically^53^.In the current study, using RNA-seq approach, tomato root gene regulation changes after long-term (48 h) application of NO donor SNP was studied. In order to find NO-responsive genes under hypoxia, common differentially regulated genes with similar regulation changes (being up- or down-regulated) under both hypoxia and SNP-treatment were identified. This study provides the potential targets of hypoxia-induced NO. The identified genes can serve as candidates for investigating their role in NO-mediated hypoxia tolerance in tomato as well as other crops.

## Result

### SNP-treatment showed distinct response in physiological parameters as well as in modulation of gene expression

**Figure 1:**
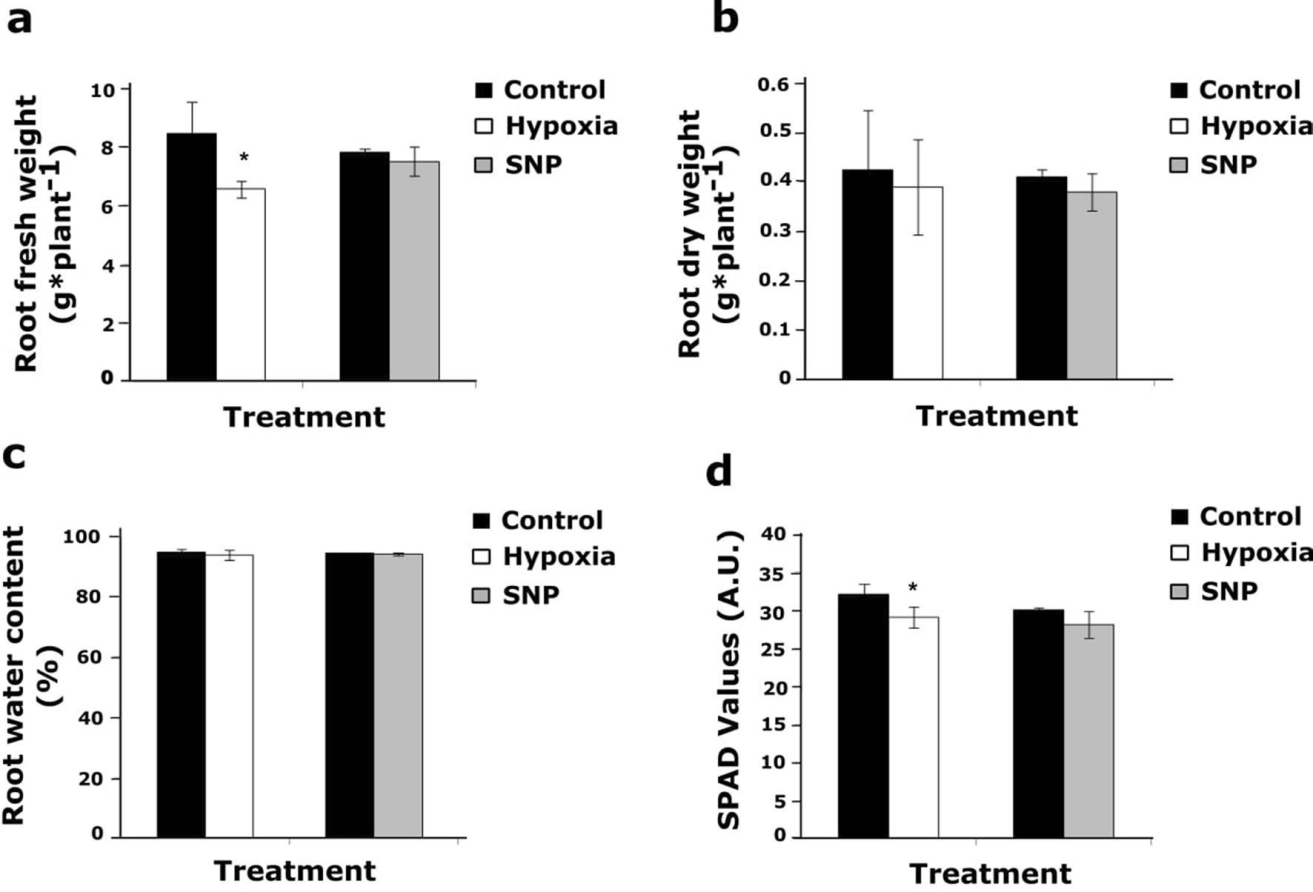
Fresh weight, dry weight and relative chlorophyll content of tomato plants in response to hypoxia and SNP-treatment. (**a**) Fresh weight; (**b**) dry weight and (**c**) water content (%) of 5 weeks old tomato roots after 48 h hypoxia and 48 h SNP-treatment were compared to their representative control. (**d**) Relative chlorophyll contents in leaf #3 of plants under hypoxia and SNP-treatment in comparison to their respective control are shown as SPAD values. Data represent means ± SD; *n* = *3*; *significant differences (Student’s t test, *P < 0.05).

In contrast to SNP-treatment, only the 48 h hypoxia resulted in a significantly lower root fresh weight (Fig. 1a). Moreover, neither hypoxia nor SNP-treatment resulted in statistically significant differences in root dry weight and root water content (%) (Fig. 1b,c). Relative chlorophyll level (SPAD values) in leaf #3showed a significant (P < 0.05) reduction in response to 48 h hypoxia, but not to SNP-treatment (Fig. 1d).

### High throughput sequencing of root RNA samples

High throughput sequencing results of 6 samples, including SNP-treated and untreated control are summarized in Table 1. Preprocessing steps, adapter clipping and low base quality filtering, resulted in about 98 and 107 million high quality reads in total (ca. 33 and 36 million reads per sample) for SNP-treated and untreated control, respectively. In total, ca. 90 and 100 million reads (91% and 93% for each sample) for untreated control and SNP-treated samples were mapped on to tomato reference genome (ITAG2.4), using Genomics workbench V7.5.5.

**Table 1:** Mapping statistics of RNA-Seq.

Sample number	Treatment	Sample ID	Total reads	Mapped reads	Mapping rate (%)	Unique match	Multi-position match
1	Without SNP	P1-minus-SNP-CR1_R1_clipped	33,693,736	30,968,120	91.91	9,927,872	21,040,248
2	Without SNP	P1-minus-SNP-CR2_R1_clipped	43,037,560	40,492,263	94.09	12,851,567	27,640,696
3	Without SNP	P1-minus-SNP-CR3_R1_clipped	21,347,691	18,634,035	87.29	5,914,647	12,719,388
4	With SNP	P1-plus-SNP-CR1_R1_clipped	31,305,704	29,155,497	93.13	9,138,316	20,017,181
5	With SNP	P1-plus-SNP-CR2_R1_clipped	39,796,110	36,633,612	92.05	11,479,837	25,153,775
6	With SNP	P1-plus-SNP-CR3_R1_clipped	36,750,959	34,754,257	94.57	10,864,379	23,889,878
Total_minus-SNP			98,078,987	90,094,418		28,694,086	61,400,332
Average_minus-SNP			32,692,996	30,031,473	91.10	9,564,695	20,466,777
Total_plus-SNP			107,852,773	100,543,366		31,482,532	69,060,834
Average_plus-SNP			35,950,924	33,514,455	93.25	10,494,177	23,020,278

Samples represent three SNP-treated and three untreated control.

### Differentially expressed genes (DEGs) in response to hypoxia and SNP-treatment (functional classification of NO and hypoxia-responsive genes)

**Figure 2:**
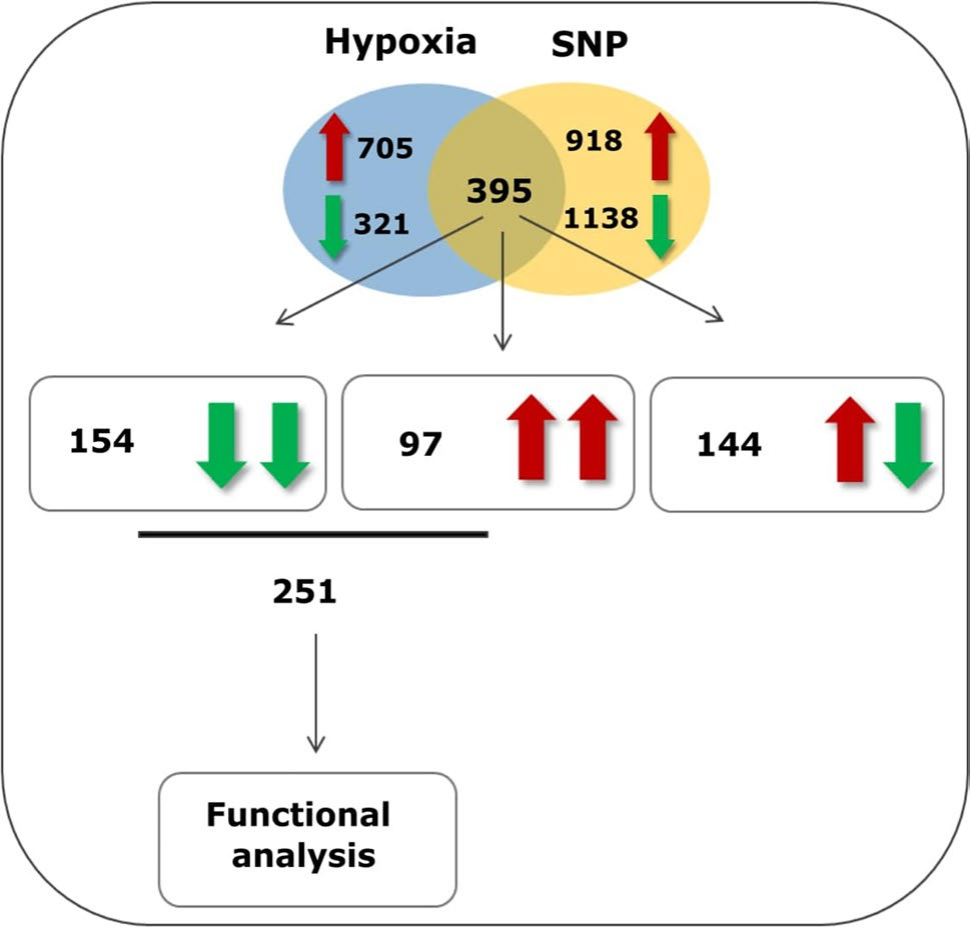
Venn diagram of common differentially expressed genes (DEGs) (Padj < 0.05) under both hypoxia and SNP-treatment. Red and green arrows represent down- and up-regulated genes, respectively. Boxes containing double green and double red arrows indicates number of down (154)- and up-regulated (97) DEGs under both hypoxia and SNP-treatments. The box containing both red and green arrows indicates the number of DEGs (144) with opposite regulation in response to hypoxia and SNP-treatment.

The number of 1144 genes were differentially regulated (Padj < 0.05) in response to 48 h SNP-treatment (792 down- and 352 up-regulated genes). After comparison with 1421 differentially regulated genes (897 up- and 524 down-regulated) under 48 h hypoxia (Padj < 0.05)^53^, it was observed that 395 DEGs, were concertedly regulated under both, hypoxia and SNP-treatment (Fig. 2). Among above-mentioned genes, 144 genes showed the opposite- while 251 genes showed similar regulation changes (154 up- and 97 down-regulated) (Fig. 2). For further analysis, only those 251 genes with similar regulation changes under NO and hypoxia were chosen for downstream biological pathway analysis. The list of all common differentially regulated genes between hypoxia and SNP-treatment is provided in Supplementary Table S1.

### Gene Ontology (GO) analysis

**Figure 3:**
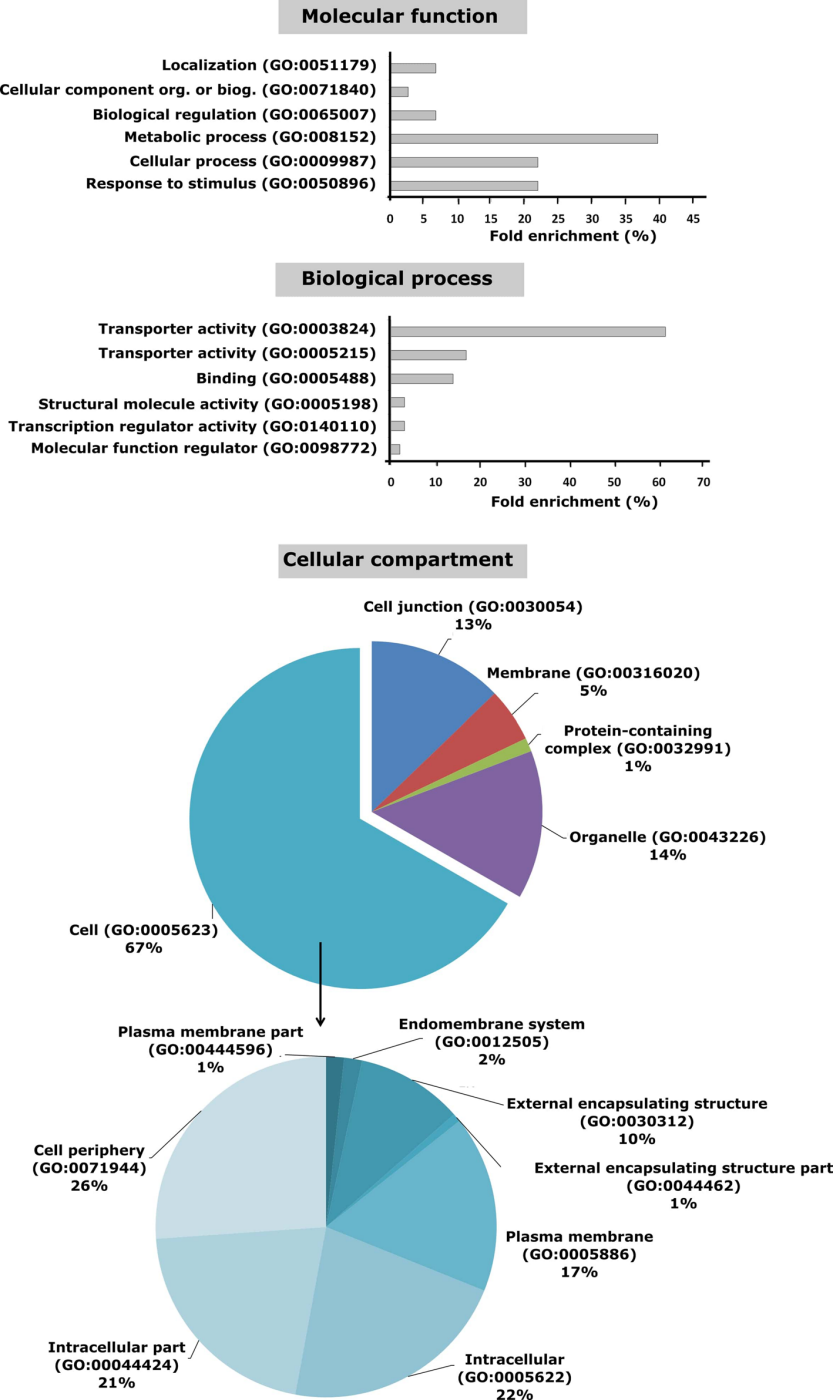
GO terms associated with transcriptome modulation of tomato roots in response to hypoxia and SNP-treatment. Enriched GO terms (*P*adj < 0.05), describing molecular function, biological process and cellular compartment. The regulated genes in all samples were analyzed for enriched GO terms using online tool PANTHER 14.0 and *Solanum lycopersicum* as a reference organism. The bars represent all significantly enriched GO terms associated with regulated genes in response to hypoxia and SNP-treatment.

To visualize enriched regulated GO terms, all significantly enriched (Padj < 0.05) Gene Ontology (GO) annotations, according to cellular component, biological processes and molecular function are presented (Fig. 3). It must be noted that GO terms refer to the proteins encoded by the genes and therefore in some cases, the word activity is used in GO term results.

Functional annotation of the regulated genes showed that their encoded proteins are mainly involved in catalytic activity (GO:0003824) (> 61%), transporter activity (GO:0005215) (17%) and binding (GO:0005488) (14%). Biological processes such as metabolic process (GO:0008152) (40%), response to stimulus (GO:0050896) (22%) and cellular process (GO:0009987) (22%) showed the highest percentage of regulated genes. The cellular component categories with the highest percentage of regulated genes were cell (GO:0005623) (67%), organelle (GO:0043226) (14%) and cell junction (GO:0030054) (13%) (Fig. 3). For a more detailed analysis of the biological pathways, we used a plant based database, MapMan^54^. MapMan categories^54^ are based on ITAG2.3 annotations (Supplementary Table S1).

### Validation of differentially expressed genes in response to SNP-treatment using qPCR

**Figure 4:**
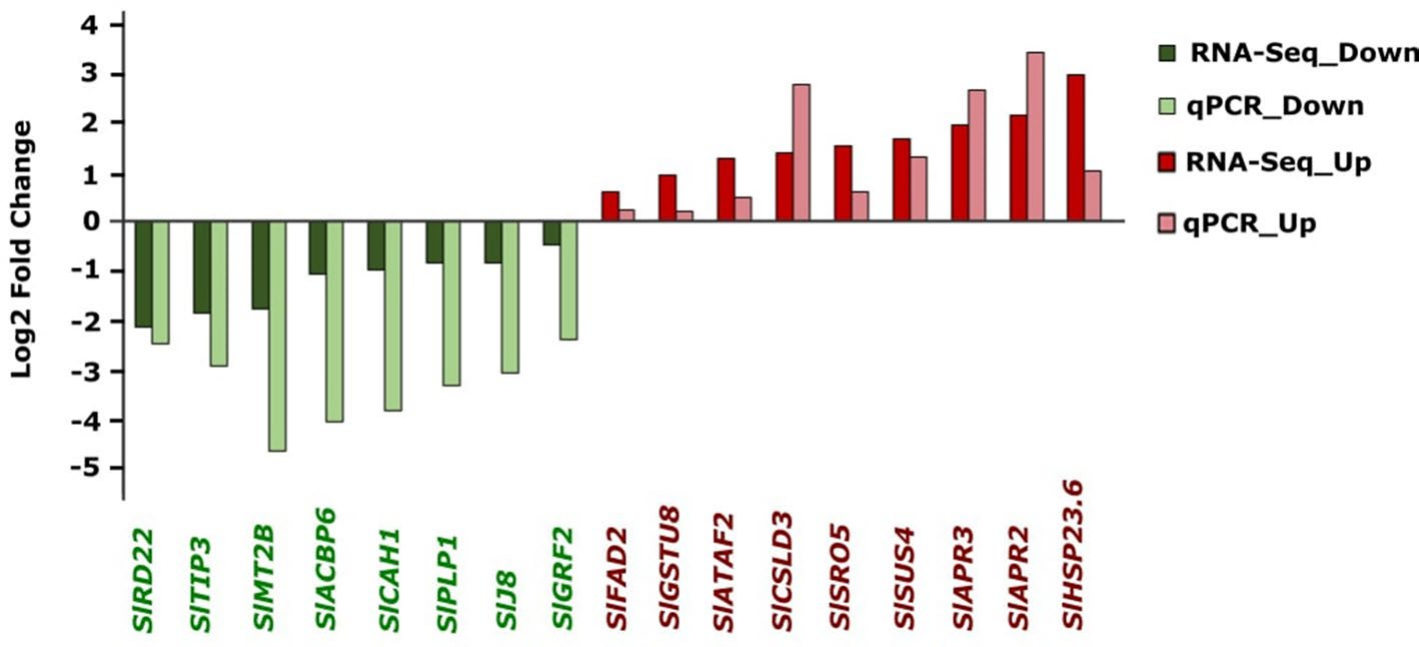
Validation of RNA-Seq data using qPCR. Log2 fold change in expression of 17 differentially regulated genes (Padj < 0.05) using RNA-Seq and qPCR (r = 0.87). Fold-changes represent the expression changes of each gene in response to SNP-treatment relative to the untreated control (n = 3).

To validate RNA-Seq data, qPCR was performed on 17 regulated genes in response to SNP-treatment (Supplementary Table S2). We observed similar gene regulation changes (SNP-treated/control) between RNA-Seq and qPCR (Fig. 4). These data confirm the validity of the RNA-Seq results used in this study. The confirmation of RNA-Seq data under hypoxia is provided elsewhere^53^.

### Hypoxia and SNP-associated phytohormone related genes

**Figure 5:**
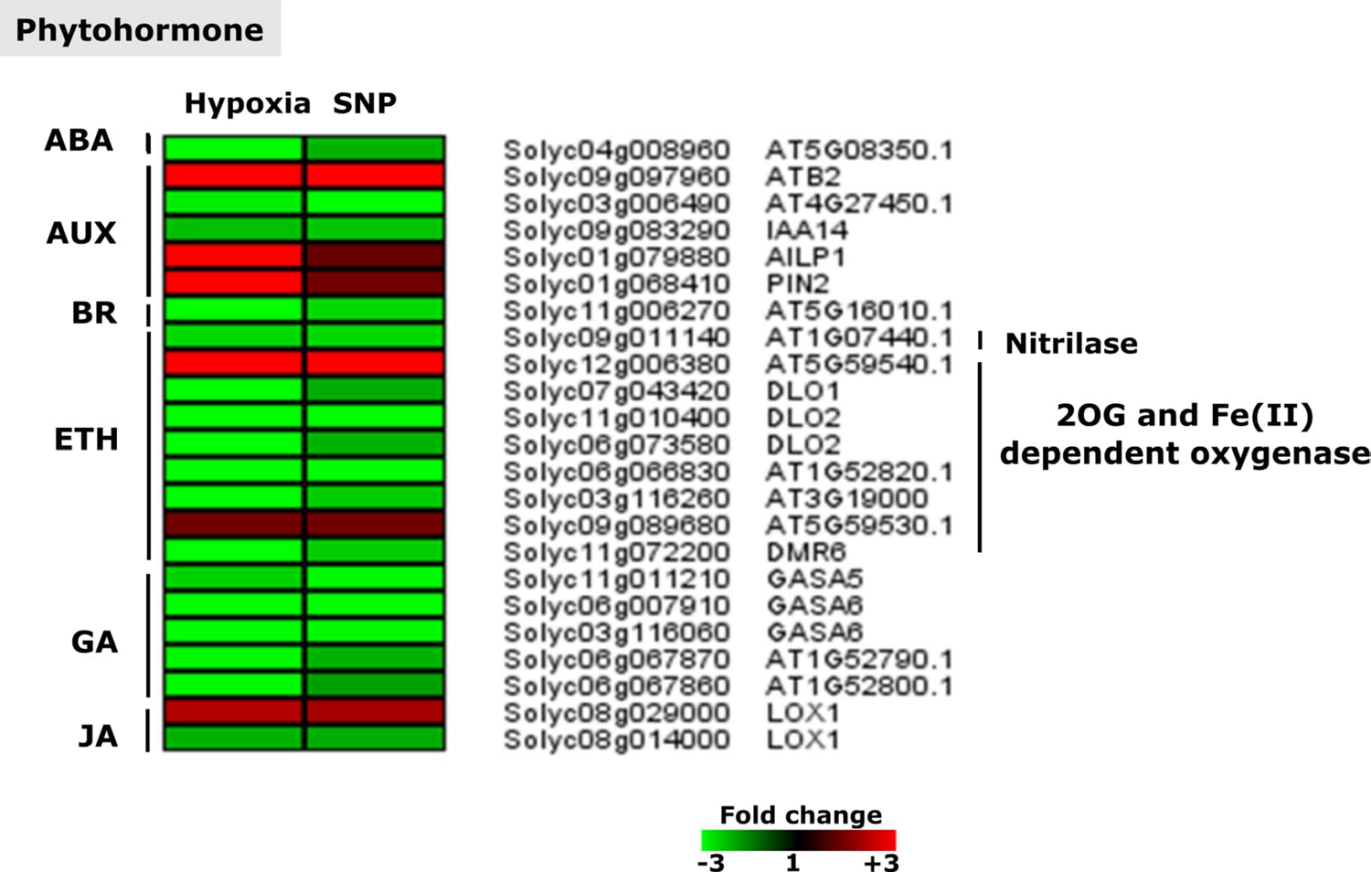
Transcriptional changes of genes related to different phytohormone categories. The heat map displays the up-regulated (red bars) or down-regulated (green bars) tomato genes and their *Arabidopsis thaliana* homologs in response to hypoxia and SNP-treatment. Depicted are differentially expressed genes (*Padj* < 0.05) (*n* = 3). ABA, abscisic acid; AUX, Auxin; BR, brassinosteroid; ETH, ethylene; GA: gibberellin; JA, jasmonate; 2OG, 2-oxoglutarate; ATB2, NAD(P)-LINKED OXIDUREDUCTASE-LIKE PROTEIN;* IAA14*,* INDOLE-3-ACETIC ACID INDUCIBLE 14*;* AILP1*,* ALUMINIUM INDUCED PROTEIN WITH YGL AND LRDR MOTIFS*;* PIN2*,* PIN-FORMED 2*;* DLO1*,* DMR6-LIKE OXYGENASE 1*;* DLO2*,* DMR6-LIKE OXYGENASE 2*;* DMR6*,* DOWNY MILDEW RESISTANT 6*;* GASA5*,* GAST1 PROTEIN HOMOLOG 5*;* GASA6*,* GAST1 PROTEIN HOMOLOG 5*;* LOX1*,* LIPOXYGENASE 1*.

It was observed that 23 hypoxia-induced phytohormone related genes responded to SNP-treatment. These genes were related to different phytohormone categories such as abscisic acid (Solyc04g008960), auxin (*ATB2*, Solyc03g006490, IAA14, *AILP1* and *PIN2*), brassinosteroid (Solyc11g006270), ethylene (Solyc12g006380, *DLO1*, two transcripts annotated as *DLO2*,* DMR6*, Solyc06g066830, Solyc03g116260, Solyc09g089680, Solyc06g073580 as well as two nitrilase encoded transcripts *MES3* and Solyc09g011140), gibberellin (*GASA5*, two transcripts annotated as GASA6 and Solyc06g067860), and jasmonate (two transcripts annotated as *LOX1*) (Fig. 5).

### Hypoxia and SNP-responsive genes encoding proteins involved in PTM and regulation of transcription (TFs)

**Figure 6:**
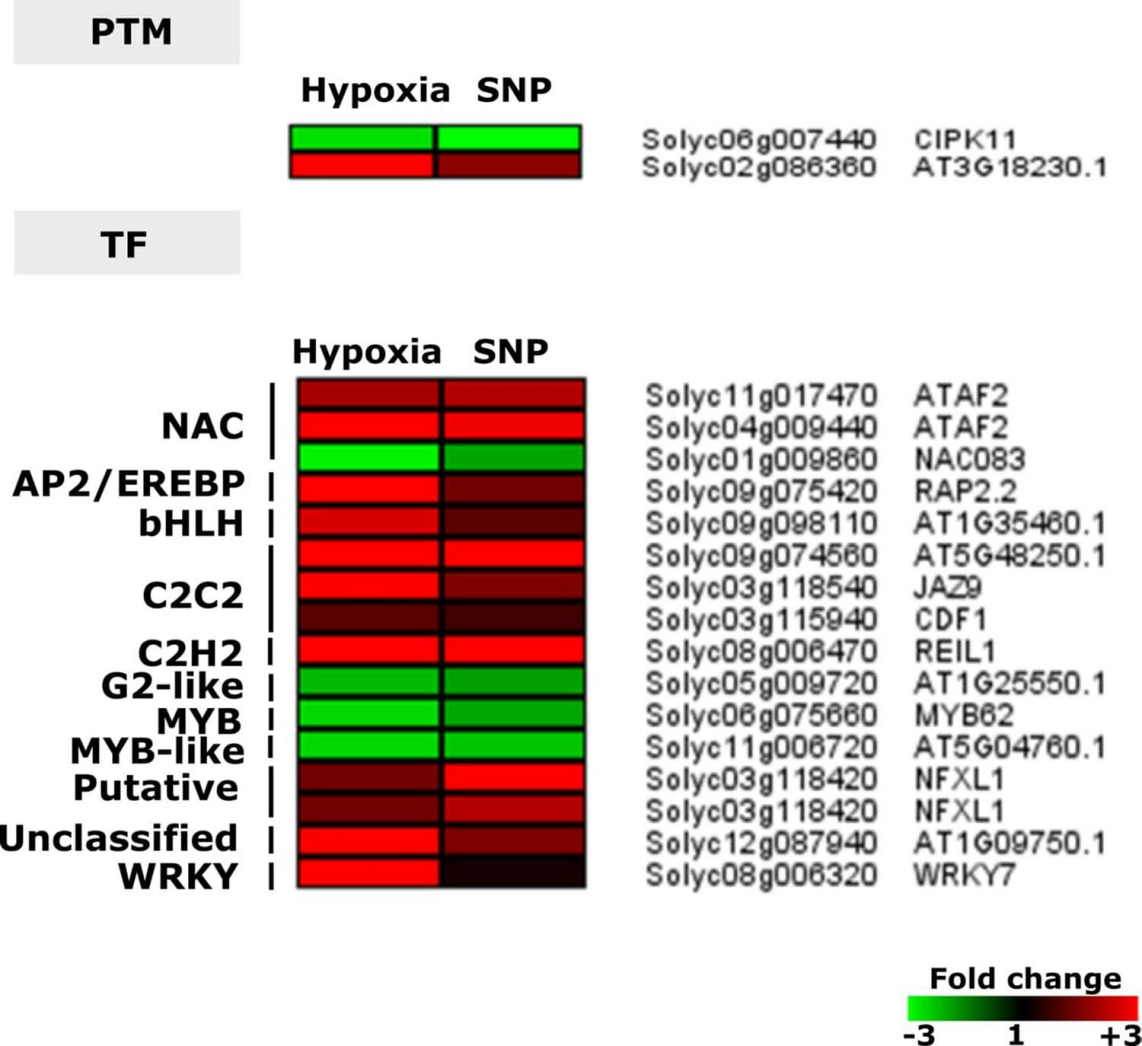
Hypoxia and SNP-responsive genes encoding proteins involved in post-translational modification (PTM) and regulation of transcription (TFs). Heat map represents the up-regulated (red bars) or down-regulated (green bars) tomato genes and their *Arabidopsis thaliana* homologs in response to hypoxia and SNP-treatment. Depicted are differentially expressed genes (*Padj* < 0.05), (*n* = 3). *CIPK11*,* CBL-INTERACTING PROTEIN KINASE 11*;* ATAF2 (NAC081)*, *ARABIDOPSIS NAC DOMAIN CONTAINING PROTEIN 81*; NOC083, *ARABIDOPSIS NAC DOMAIN CONTAINING PROTEIN 83*;* RAP2.2*,* RELATED TO AP2 2*;* JAZ9*,* RELATED TO AP2 2*;* CDF1*,* CYCLING DOF FACTOR 1*;* REIL1*,* REI1-LIKE 1*;* MYB62*,* MYB DOMAIN PROTEIN 62*;* NFXL1*,* NF-X-LIKE 1*;* WRKY7*,* WRKY DNA-BINDING PROTEIN 7*.

Two genes encoding proteins involved in PTM (*CIPK11* and Solyc02g086360) showed regulation changes in response to hypoxia and SNP-treatment. Moreover, 16 genes encoding members of different transcription factor (TF) families were observed, as follow: NAC family (two transcripts annotated as *ATAF2/NAC081* and one transcript as *NAC083*), AP2/EREBP (*RAP2.2*), Aux/IAA family (*IAA14*), *bHLH* (Solyc09g098110), C2C2 (Solyco9g074560, *JAZ9* and *CDF1*), C2H2 zinc finger family(*REIL1*), G2-like (Solyc05g009720), MYB (*MYB62)*,* MYB-like* (Solyc11g006720), WRKY (*WRKY7*), one transcript annotated as unclassified (Solyc12g087940) and two transcripts encoding a putative transcription factor (*NFXL1*) (Fig. 6).

### Common DEGs involved in primary metabolism

**Figure 7:**
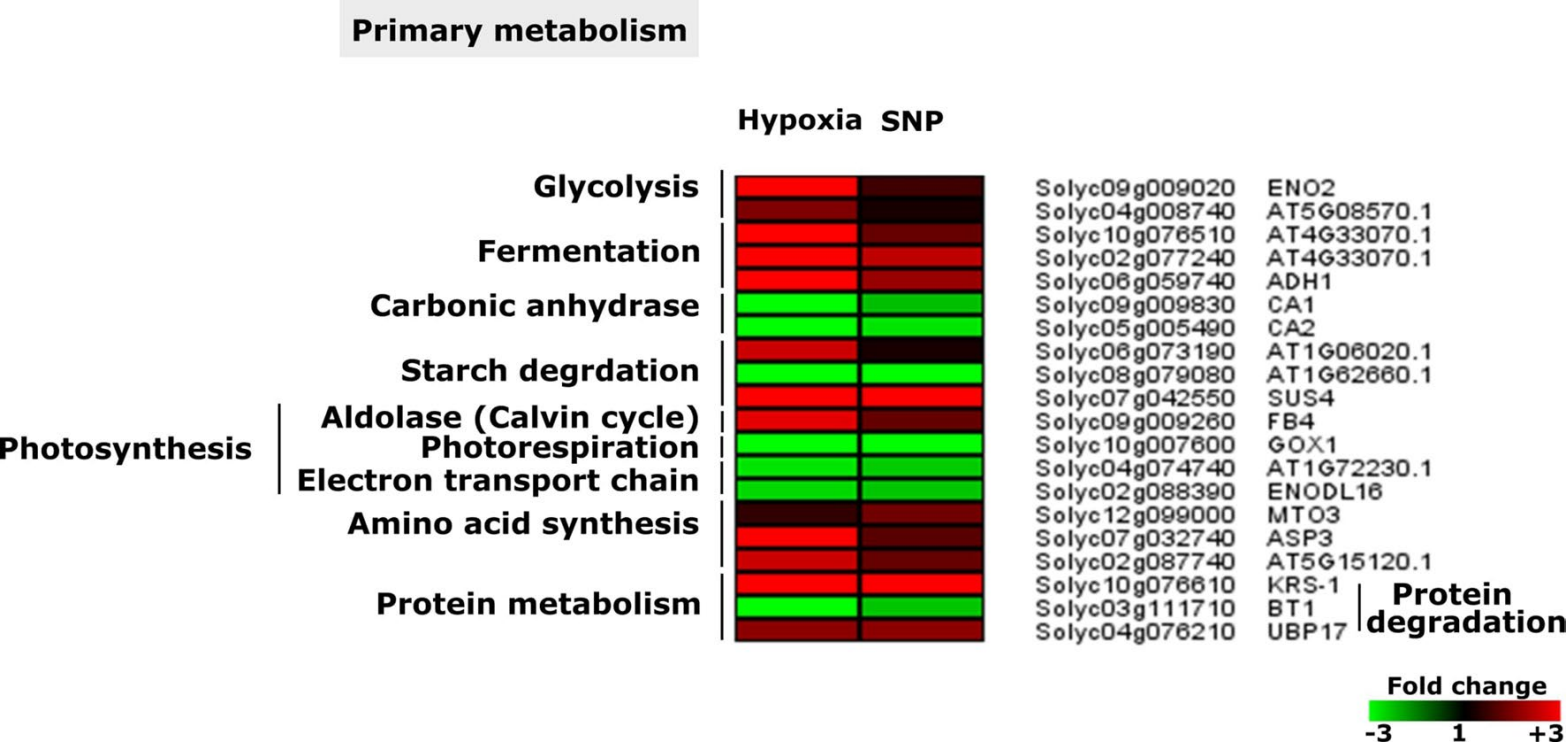
Hypoxia- and SNP-induced and repressed genes associated with primary metabolism. Heat maps display the up-regulated (red bars) or down-regulated (green bars) tomato genes and their *Arabidopsis thaliana* homologs. Depicted are differentially expressed genes (Padj < 0.05) (n = 3*). ENO2*,* ENOLASE2*;* ADH1*,* ALCOHOL DEHYDROGENASE 1*;* CA1*,* CARBONIC ANHYDRASE 1*;* CA2*,* CARBONIC ANHYDRASE 2*;* SUS4*,* SUCROSE SYNTHASE 4*;* FB4*,* AUXIN SIGNALING F-BOX 4*;* GOX1*,* GLYCOLATE OXIDASE 1*;* ENODL16*,* EARLY NODULIN-LIKE PROTEIN 16*;* MTO3*,* METHIONINE OVER-ACCUMULATOR 3*;* ASP3*,* ASPARTATE AMINOTRANSFERASE 3*;* KRS.1*,* LYSYL-TRNA SYNTHETASE 1*;* BT1*,* BTB AND TAZ DOMAIN PROTEIN 1*;* UBP17*,* UBIQUITIN-SPECIFIC PROTEASE 17*.

Among the responsive genes to both hypoxia and NO, were the genes encoding enzymes belonging to the primary metabolism such as glycolysis (*ENO2* and Solyc04g008740), fermentation(*ADH1*, Solyc02g077240 and solyc10g076510), carbon metabolism (carbonic anhydrase: *CA1* and *CA2*), starch degradation (Solyc06g073190, Solyc08g079080 and *SUS4*), photosynthesis: Calvin cycle (*FBA4*), photorespiration (*GOX1*) and electron transport chain (Solyc04g074740 and *ENODL16*), amino acid synthesis (*MTO3*,* ASP3*,* Solyc02g087740*), protein synthesis (*BT1*) and protein degradation (*KRS-1 and UBP17*) (Fig. 7).

### Redox associated genes responsive to hypoxia and SNP-treatment

**Figure 8:**
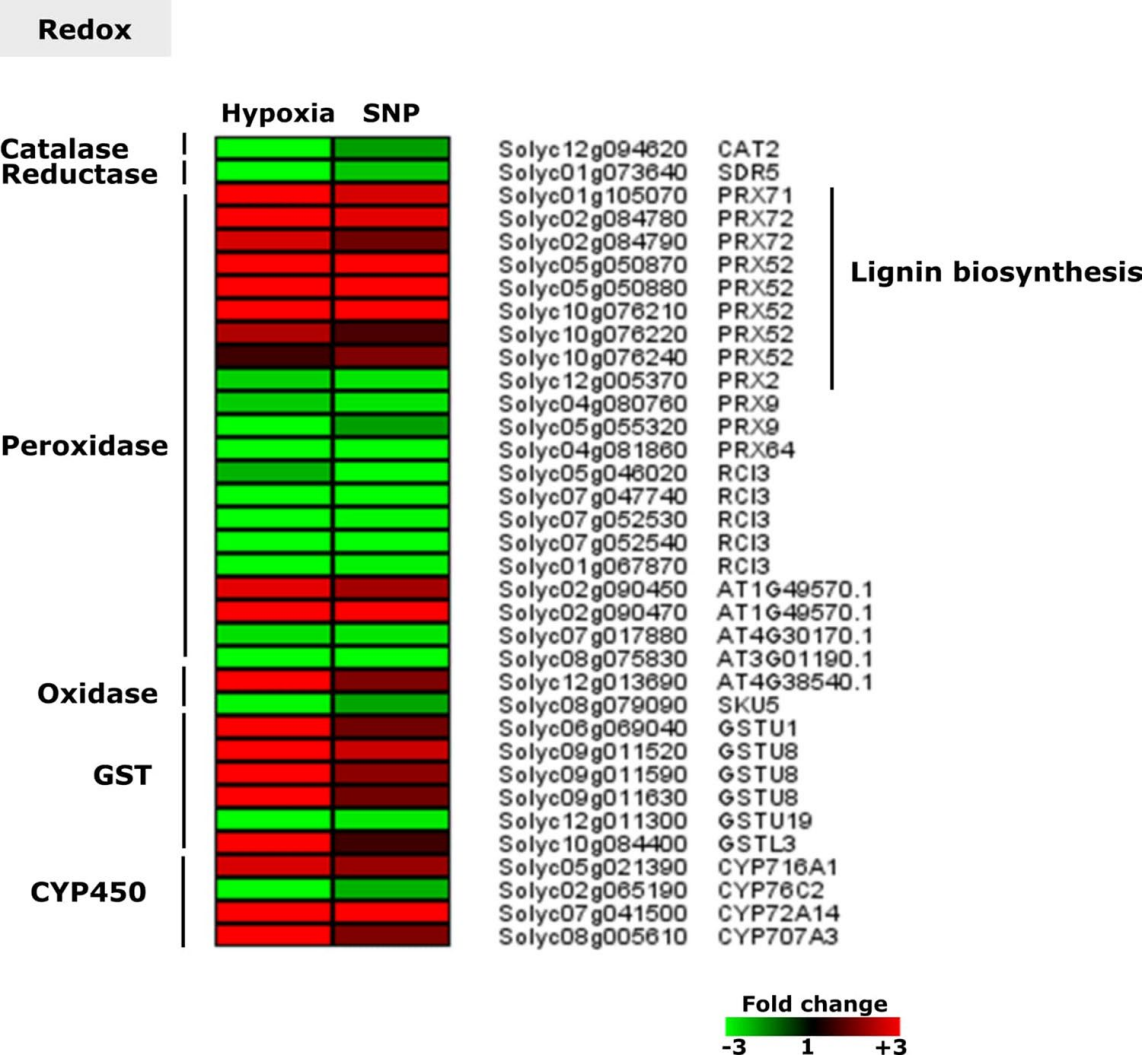
Expression pattern of genes encoding members of different antioxidant classes. Heat map represents the up-regulated (red bars) or down-regulated (green bars) tomato genes and their *Arabidopsis thaliana* homologs in response to hypoxia and SNP-treatment. Depicted are differentially expressed genes (*Padj* < 0.05), (*n* = 3). *GST*,* GLUTATHIONE S-TRANSFERASE*; *CAT2*,* CATALASE 2*;* SDR5*,* SHORT-CHAIN DEHYDROGENASE REDUCTASE 5*;* PRX71*,* PEROXIDASE 71*;* PRX72*,* PEROXIDASE 72*;* PRX52*,* PEROXIDASE 52*;* PRX2*,* PEROXIDASE 2*;* PRX9*,* PEROXIDASE 9*;* PRX64, PEROXIDASE 64*;* RCI3*,* RARE COLD INDUCIBLE GENE 3*;* SKU5*;* encodes SKU5 protein*;* GSTU1 (GST19)*,* GLUTATHIONE S-TRANSFERASE TAU 1 (GLUTATHIONE S-TRANSFERASE 19)*;* GSTU8*,* GLUTATHIONE S-TRANSFERASE TAU 8*;* GSTU19 (GST8)*,* GLUTATHIONE S-TRANSFERASE TAU 19 (GLUTATHIONE S-TRANSFERASE 8)*;* GSTL3*,* encoding a member of glutathione S-transferase family protein*;* CYP716A1*,* CYTOCHROME P450*,* FAMILY 716*,* SUBFAMILY A*,* POLYPEPTIDE 1*;* CYP76C2*,* CYTOCHROME P450*,* FAMILY 76*,* SUBFAMILY C*,* POLYPEPTIDE 2*;* CYP72A14*,* CYTOCHROME P450*,* FAMILY 72*,* SUBFAMILY A*,* POLYPEPTIDE 14*;* CYP707A3*,* CYTOCHROME P450*,* FAMILY 707*,* SUBFAMILY A*,* POLYPEPTIDE 3*.

Among regulated genes under hypoxia and SNP-treatment, 35 genes were observed encoding redox-associated proteins belonging to different categories such as catalases (*CAT2*), reductases (*SDR5*), peroxidases (*PRX71*, two transcripts annotated as *PRX72*, five transcripts annotated as *PRX52*, *PRX2*, two transcripts annotated as *PRX9*, *PRX64*, five transcripts annotated as *RCI3*, Solyc02g090450, Solyc02g090470, Solyc07g017880 and Solyc08g075830, oxidases (Solyc12g013690 and *SKU5*), glutathione S-transferases (*GSTU1*, three transcripts annotated as *GSTU8*, *GSTU19* and *GSTL3*), cytochrome P450 (*CYP716A1*, *CYP76C2*, *CYP72A14* and *CYP707A3*) (Fig. 8). These data indicate the importance of redox regulation under hypoxia-induced nitrosative stress.

### Concertedly regulated DEGs associated with cellular transport

**Figure 9:**
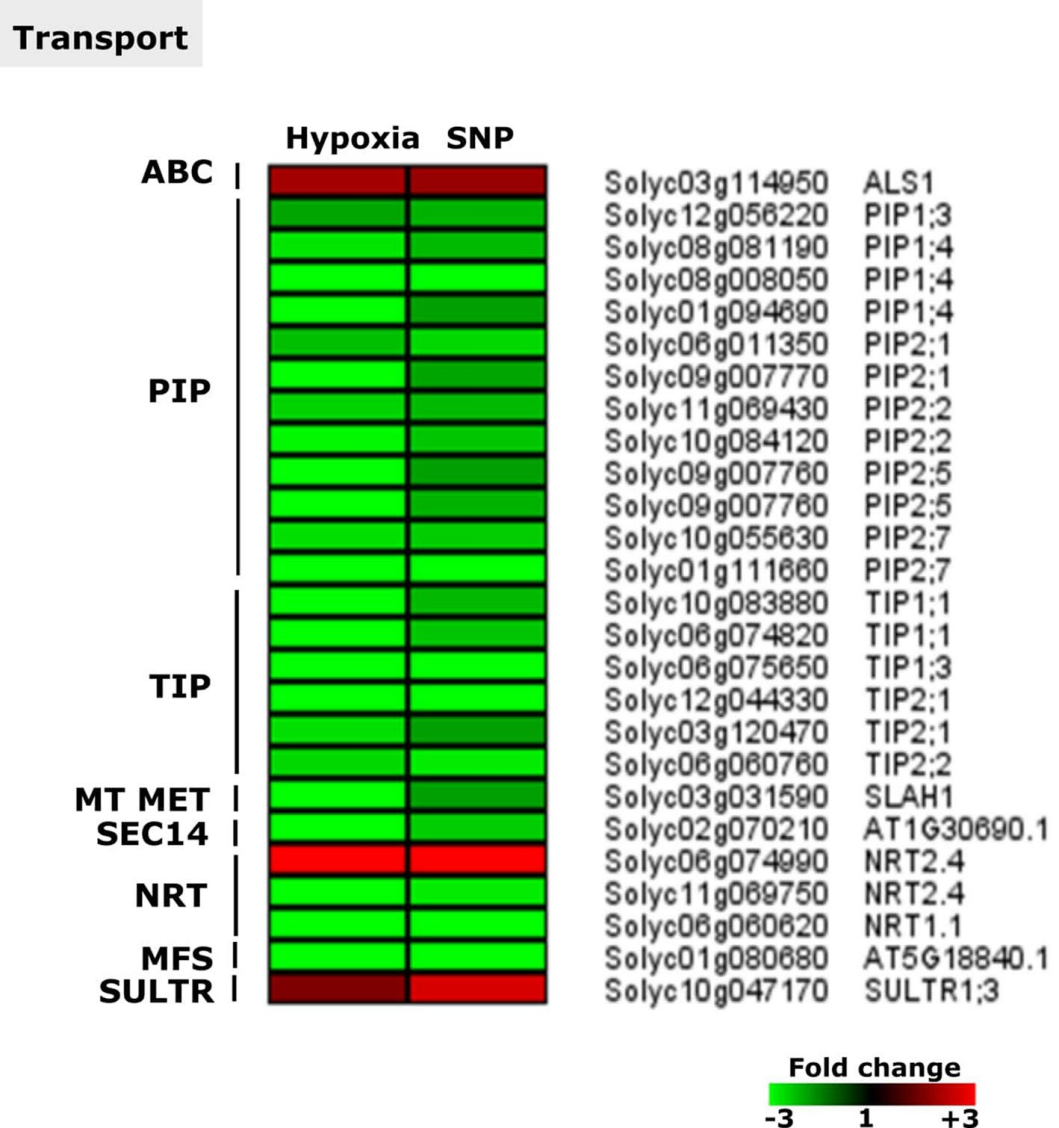
Expression pattern of genes related to cellular transport. Heat map represents the up-regulated (red bars) or down-regulated (green bars) tomato genes and their *Arabidopsis thaliana* homologs in response to hypoxia and SNP-treatment. Depicted are differentially expressed genes (*Padj* < 0.05), (*n* = 3). ABC, ATP-binding cassette transporter; PIP, major intrinsic proteins; TIP, tonoplast intrinsic protein; MT MET, metabolite transporters at the mitochondrial membrane, SEC14, Sec14p-like phosphatidylinositol transfer family; NRT, nitrate transporter; MFS, major facilitator superfamily; SULTR, sulfur transporter. *ALS1*,* ALUMINUM SENSITIVE 1*;* PIP1*;*3*,* PLASMA MEMBRANE INTRINSIC PROTEIN 1*;*3*;* PIP1*;*4*,* PLASMA MEMBRANE INTRINSIC PROTEIN 1*;*4*;* PIP2*;*1*,* PLASMA MEMBRANE INTRINSIC PROTEIN 2*;*1*;* PIP2*;*2*,* PLASMA MEMBRANE INTRINSIC PROTEIN 2*;*2*;* PIP2*;*5*,* PLASMA MEMBRANE INTRINSIC PROTEIN 2*;*5*;* PIP2*;*7*,* PLASMA MEMBRANE INTRINSIC PROTEIN 2*;*7*;* TIP1*;*1*,* TONOPLAST INTRINSIC PROTEIN 1*;*1*;* TIP1*;*3*,* TONOPLAST INTRINSIC PROTEIN 1*;*3*;* TIP2*;*1*,* TONOPLAST INTRINSIC PROTEIN 2*;*1*;* TIP2*;*2*,* TONOPLAST INTRINSIC PROTEIN 2*;*2*;* SLAH1*,* SLAC1 HOMOLOGUE 1*;* NRT2.4*,* NITRATE TRANSPORTER 2.4*;* NRT1.1*,* NITRATE TRANSPORTER 1.1*;* SULTR1.3*,* SULFATE TRANSPORTER 1.3*.

Among common DEGs under hypoxia and SNP-treatment, 27 genes were observed encoding proteins related to transport (3 up- and 17 down-regulated). These genes encode proteins involved in the transport of sulfate (*SULTR1*;*3*), peptides and oligopeptides (*NRT1.1*) and nitrate (two transcripts encoding NRT2.4). Furthermore, a member of Sec14p-like phosphatidylinositol transfer family(solyc02g070210), an ATP-binding cassette(ABC) transporter (*ALS1*), mitochondria membrane metabolite transporter (*SLAH1*), a sugar transporter (Solyc01g080680) and major intrinsic proteins, aquaporins ( AQP), such as 12 plasma membrane intrinsic proteins (PIPs: *PIP1*;*3*, three transcripts annotated as *PIP1*;*4* (three transcripts), *PIP2*;*1* (two transcripts), *PIP2*;*2*, *PIP2*;*5* (two transcripts) and *PIP2*;*7* (two transcripts). Among the regulated genes were also 7 tonoplast intrinsic proteins (TIPs: *TIP1*;*1* (two transcripts), *TIP1*;*3*, *TIP2*;*1* (two transcripts), *TIP2*;*2* and major facilitator superfamily (MSF: Solyc01080680) were identified (Fig. 9).

### DEGs associated with biotic and abiotic stress

**Figure 10:**
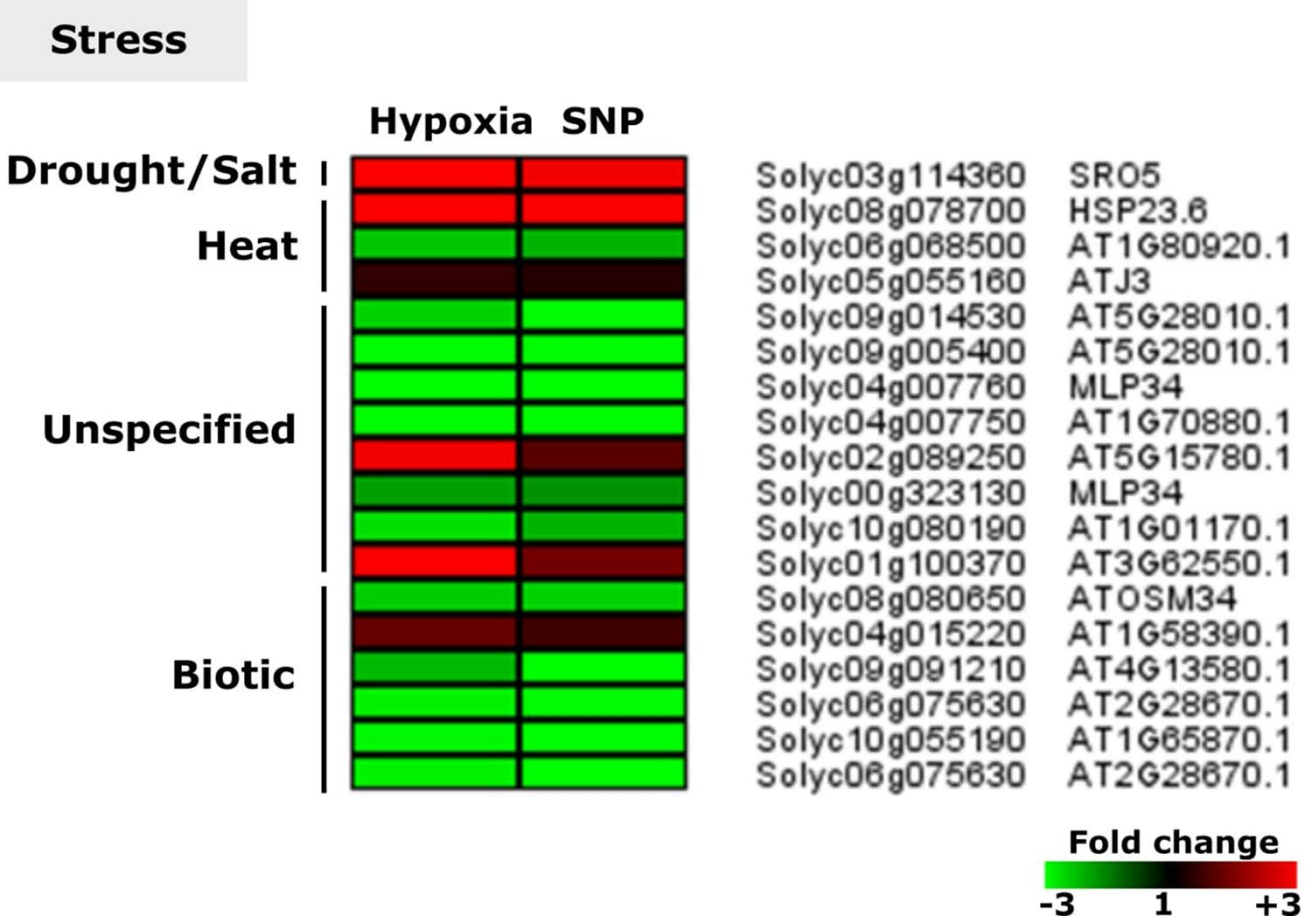
Expression pattern of biotic and abiotic stress related genes. Heat map represents the up-regulated (red bars) or down-regulated (green bars) tomato genes and their *Arabidopsis thaliana* homologs in response to hypoxia and SNP-treatment. Depicted are differentially expressed genes (*Padj* < 0.05), (*n* = 3). *SRO5*,* SIMILAR TO RCD ONE 5*;* HSP23.6*,* MITOCHONDRION-LOCALIZED SMALL HEAT SHOCK PROTEIN 23.6*;* ATJ3*,* DNAJ HOMOLOGUE 3*;* MLP34*,* MLP-LIKE PROTEIN 34*;* OSM34*,* OSMOTIN 34*.

Out of 19 stress related genes, which were responsive to hypoxia and SNP-treatment, six were related to biotic stress, encoding PR genes (Solyc08g080650, Solyc04g015220, Solyc09g091210, Solyc06g075630, Solyc10g055190 and Solyc06g075630). 13 genes were identified being related to abiotic stresses such as drought/salt (two transcripts annotated as *SRO5*), heat (*HSP23.6*, Solyc06g068500 and *ATJ3*) and an unspecified class of stress (Solyc09g014530, Solyc09g005400, *MLP34*, Solyc04g007750, Solyc10g080190 and Solyc01g100370). The rest of the abiotic stress related genes (*OSM34*, Solyc04g015220, Solyc09g091210, Solyc06g075630, Solyc10g055190 and Solyc06g075630) were unspecified (Fig. 10).

### Cell wall related genes regulated in response to hypoxia and SNP-treatment

**Figure 11:**
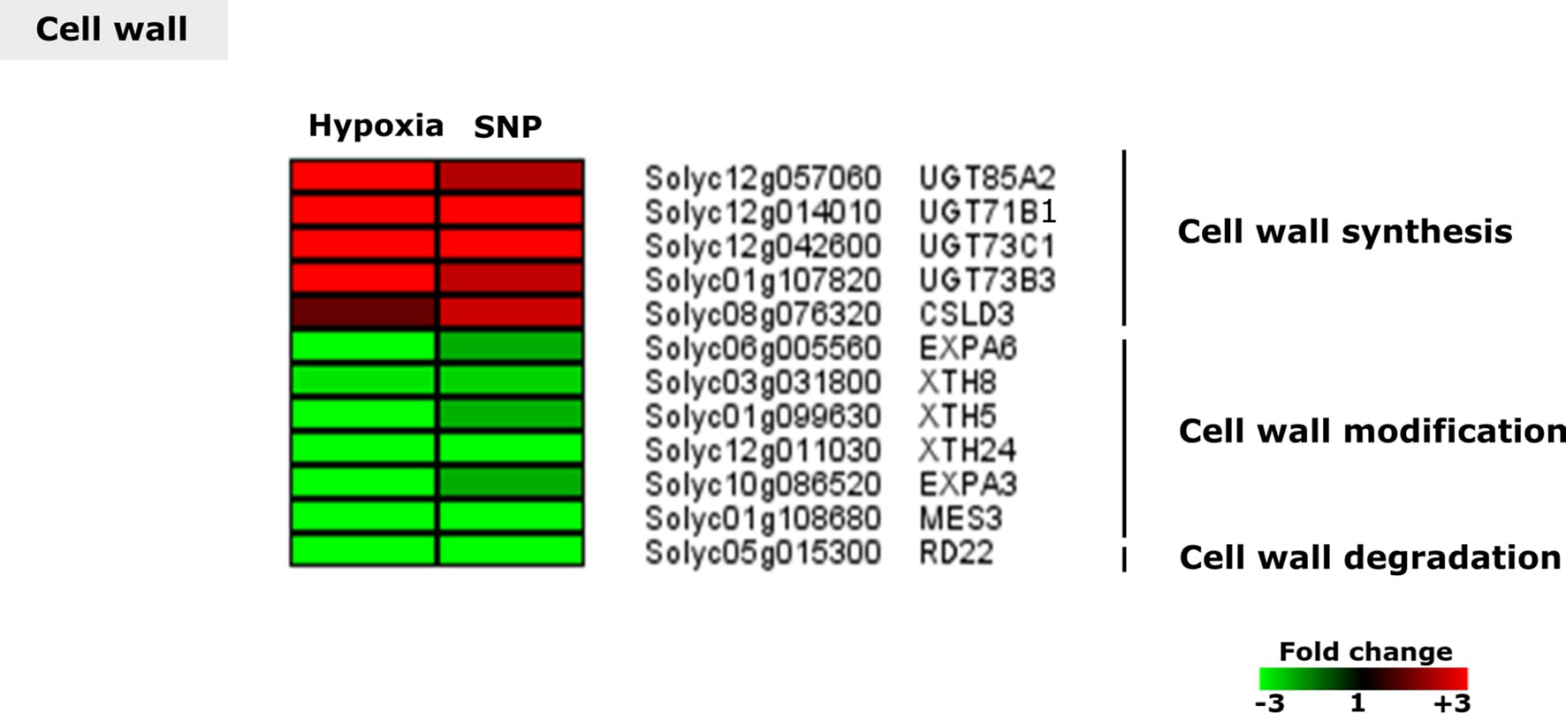
Expression pattern of regulated cell wall related genes. Heat map represents the up-regulated (red bars) or down-regulated (green bars) tomato genes and their *Arabidopsis thaliana* homologs in response to hypoxia and SNP-treatment. Depicted are differentially expressed genes (*Padj* < 0.05), (*n* = 3). *UGT85A2*,* UDP-GLUCOSYL TRANSFERASE 85A2*;* UGT71B1*,* UDP-GLUCOSYL TRANSFERASE 71B1*;* UGT73C1*,* UDP-GLUCOSYL TRANSFERASE 73C1*;* UGT73B3*,* UDP-GLUCOSYL TRANSFERASE 73B3*;* CSLD3*,* CELLULOSE SYNTHASE LIKE D3*;* RD22*,* RESPONSIVE TO DESICCATION 22*;* EXPA6*,* EXPANSIN 6*;* XTH8*,* XYLOGLUCAN ENDOTRANSGLUCOSYLASE/HYDROLASE 8*;* XTH5*,* XYLOGLUCAN ENDOTRANSGLUCOSYLASE/HYDROLASE 5*;* XTH24*,* XYLOGLUCAN ENDOTRANSGLUCOSYLASE/HYDROLASE 24*;* EXPA3*,* EXPANSIN 3*;* MES3*,* METHYL ESTERASE 3*.

Several cell wall related genes showed regulation changes in response to hypoxia and SNP-treatment. These transcripts were related to cell wall synthesis (*CSLD3*,* UGT85A2*, *UGT71B1*, *UGT73C1* and *UGT73B3*), Cell wall modification (*XTH8*, *XTH5* and *XTH24*,* EXPA6* and *EXPA3* ) and cell wall degradation (*RD22*) (Fig. 11).

### Other biological pathways

**Figure 12:**
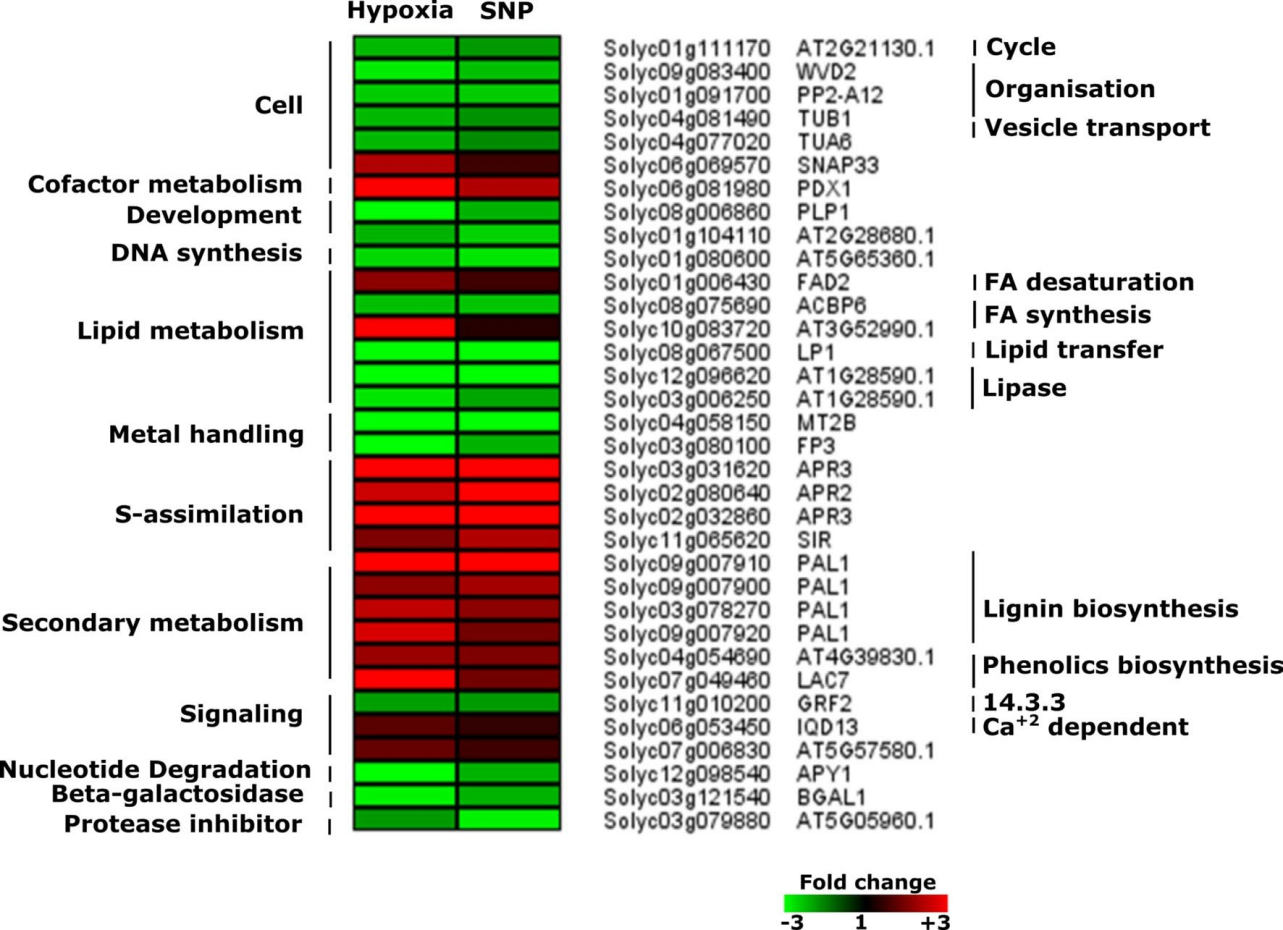
Expression pattern of genes belonging to different biological categories. Heat map represents the up-regulated (red bars) or down-regulated (green bars) tomato genes and their *Arabidopsis thaliana* homologs in response to hypoxia and SNP-treatment. Depicted are differentially expressed genes (*Padj* < 0.05), (*n* = 3). *WVD2*,* WAVE-DAMPENED 2*;* PP2-A12*,* PHLOEM PROTEIN 2-A12*,* TUB1*,* TUBULIN BETA-1 CHAIN*;* TUA6*,* TUBULIN ALPHA-6*;* SNAP33*,* SOLUBLE N-ETHYLMALEIMIDE-SENSITIVE FACTOR ADAPTOR PROTEIN 33*;* PDX1*,* PYRIDOXINE BIOSYNTHESIS 1*;* PLP1*,* PHOSPHOLIPASE 1*;* FAD2*,* FATTY ACID DESATURASE 2*;* ACBP6*,* ACYL-COA-BINDING PROTEIN*;* LP1*,* LIPID TRANSFER PROTEIN 1*;* MT2B*,* METALLOTHIONEIN 2B*;* FP3*,* FARNESYLATED PROTEIN 3*;* APR3*,* APS REDUCTASE 3*;* APR2*,* 5′ADENYLYLPHOSPHOSULFATE REDUCTASE 2*;* SIR*,* SULFITE REDUCTASE*;* PAL1*,* PHENYLALANINE AMMONIA-LYASE*;* LAC7*,* LACCASE 7*;* GRF2*,* GENERAL REGULATORY FACTOR 2*;* IQD13*,* IQ-DOMAIN 13*;* APY1*,* APYRASE 1*;* BGAL1*,* BETA GALACTOSIDASE 1*.

Differentially regulated genes responsive to hypoxia and SNP-treatment encoding proteins belonging to additional functional categories were identified: lipid metabolism (*LP1*, *ACBP6*, *FAD2* and Solyc10g083720) cell cycle (solyc01g111170, Solyc01g111170), cell organization (*WVD2*, *PP2-A12*, *TUB1* and *TUA6*), cell vesicle transport (*SNAP33*), co-factor and vitamin metabolism (*PDX1*), development/storage proteins (*PLP1* and solyc01g104110), DNA synthesis (Solyc01g080600), metal handling (*MT2B* and *FP3*), S-assimilation (two transcripts annotated as *APR3*, *APR2* and *SIR*), secondary metabolism such as lignin (solyc04g054690 and LAC7) and phenol (three transcripts annotated as *PAL1*), Signaling (*GRF2*, *IQD1* and Solyc07g006830), nucleotide metabolism (*APY1*), beta-galactosidase (*BGAL1*) and protease inhibitor (solyc03g079880) (Fig. 12).

## Discussion

Tomato is a commercially important edible crop^10^. Improving tomato fruit size during the domestication process has been achieved by compensating its stress tolerance^55^. Former studies showed that SNP application on tomato root enhances salt stress tolerance, exhibited in improved growth and higher chlorophyll content. In the same study, SNP-treatment resulted in lower lipid oxidation, higher activity of antioxidant enzymes (SOD, APX, GR and POD) as well as an increase in ascorbate and proline content^10^. These data suggest that exogenous NO application via SNP-treatment is a reliable system for investigating the NO effect during stress response in the plant.

Several former studies investigated gene expression regulation in response to exogenous NO application using different NO donors in various plant species such as Arabidopsis^11,18,56^, cotton^57^ and birch^58^. However, our knowledge about the role of hypoxia-induced NO on gene expression modulation in tomato root is still scarce.

The current study represents the comparative transcriptome modulation of tomato (*cv.* Moneymaker) root in response to long-term (48 h) hypoxia^53^ and SNP-application. Long-term hypoxia, but not SNP-treatment, resulted in significantly (P < 0.05) lower root fresh weight and chlorophyll content (SPAD values) (Fig. 1a,d).

Under hypoxia as well as under SNP-treatment, 395 genes (28% of hypoxia-regulated genes), were concertedly regulated. Among them, 251 genes, corresponding to. 64% of common regulated genes, were similarly up- or down-regulated under both conditions (Fig. 2). It was also noticeable that the number of common down-regulated genes (154) in response to hypoxia and SNP-treatment was higher than up-regulated genes (97).

To validate the RNA-Seq. data, the expression changes of 17 differentially regulated genes were confirmed using qPCR. A high level of consistency between the qPCR and RNA-Seq expression data indicates the reliability of transcriptome data (Fig. 4).

### Phytohormone associated genes showed regulation changes response to hypoxia and SNP-treatment

Genes belonging to different phytohormonal categories such as auxin, ethylene, jasmonate and gibberellin revealed regulation changes in response to NO application and hypoxia (Fig. 5). NO modulates auxin effect on root architecture. Transcriptional regulation of auxin related genes in response to exogenous NO application has already been reported^59^. Among auxin related DEGs in response to hypoxia and SNP-treatment in our study, polar auxin transporter *PIN2* showed the highest up-regulation (12-fold) under hypoxia (Fig. 5). In Arabidopsis, ERFVII mediated repression of *PIN2*, has been shown to be associated with root bending under hypoxia in soil grown roots^60^. Since in this study tomato were cultivated in a hydroponic system, hypoxia might not cause the same response as in soil grown roots. These data suggest that polar auxin transport seems to be involved in root growth response under hypoxia. However, species-specific response, effects of growth condition as well as NO-dependent regulation of *PIN2* expression remain unclear.

*IAA14* showed down-regulation in response to hypoxia and SNP-treatment (Fig. 5). IAA14 is a negative regulator of auxin response factors ARF7 and ARF19, which are involved in lateral root initiation via induction of multiple LBD/ASLs such as LBD16/ASL18 and LBD29/ASL16^61^. It has been shown that overexpression of IAA14 leads to inhibition of lateral root formation in *Arabidopsis thaliana*^62^. Moreover, this indicates that IAA14 negatively regulates lateral root formation. This is in accordance with its downregulation in response to hypoxia, when adventive root formation is beneficial for low oxygen tolerance. Down-regulation of *IAA14 in response to SNP* in this study indicates that its regulation might be NO dependent. Recently, it has been shown that ERF VII TFs are involved in the regulation of lateral root formation through repression of auxin-induced genes (*LBD16*, *LBD18*, and *PUCHI* and *IAA19*)^63^. To what extent this process is associated with NO signaling, requires further investigation.

Regulation changes in ethylene related genes were observed in the current study (Fig. 5). Ethylene, one of the key regulators of hypoxia response, is involved in aerenchyma formation under flooding stress and has been shown to have cross talk with NO^64^. Recently, it has been reported that early ethylene-induced *PHYTOGLOBIN1* (*PGB1*) acts as NO scavenger leading to the ERFVII stability and adaption to upcoming hypoxia^65^. Moreover, investigation of *ein2-1 nos1/noa1* double mutant in *A. thaliana* revealed cross talk between ethylene, through EIN2, and NO signaling in the regulation of dark-induced leaf senescence^66^. These data indicate the importance of NO signaling and ethylene response during hypoxia.

Defense related phytohormones such as JA and SA have been shown to have an interplay with NO. In the current study, two transcripts being annotated as *LOX1 (LIPOXYGENASE 1)* showed regulation changes (Fig. 5). Mutation of *LOX1* gene in Arabidopsis has been shown to modify both signaling and redox related response under cadmium stress^67^. Former studies in *A. thaliana* confirmed the expression induction of *AOS* and *LOX2* in response to SNP application^68^. It has been shown that NO increased the expression of several JA biosynthetic genes among them *LOX3*, encoding the enzyme involved in the conversion of linolenic acid to 13(s) hydro peroxy octadecatrieonic acid^69^. Our result is in accordance with the above-mentioned studies and suggests a possible role for hypoxia-induced NO on regulation of some of the JA biosynthesis genes.

NO has been shown to play a role in regulating GA biosynthesis and signal transduction^70^. Both antagonistic and synergic interaction between NO and GA has been reported^71^. Five GA related genes showed down-regulation in our study (Fig. 3), among them, GASA5 has been reported to negatively regulate GA-induced flowering and stem growth^72^. However, its role during hypoxia in the root and its interaction with NO has not yet been investigated. Our results imply a cross talk between NO, hypoxia and different phytohormone related genes.

### NO responsive TFs play role in diverse physiological processes and stress response

Group VII ERFs (ERFVII) has been shown to play a key role in hypoxia sensing and signaling via oxygen and NO dependent N-end rule pathway^50,73,74^. NO sensing has been shown to be executed via oxidation of Cysteine residue of ERFVII TFs, followed by arginylation and ubiquitination for proteasomal degradation (N-degron pathway)^48,75^. An ERFVII member, *RAP2.2*, was significantly up-regulated in response to hypoxia and SNP-treatment (Fig. 6). It has been reported that *RAP2.2* overexpressing lines exhibited an improved hypoxia survival response while knockout lines showed weaker survival rate compared to the wild type. Moreover, RAP2.2 regulates the expression of hypoxia-responsive genes which their encoded enzymes are involved in sugar metabolism as well as fermentation pathways^76^. These data indicate the significant role of RAP2.2 in root response to hypoxia.

Moreover, among regulated genes were several transcripts (*DLO1*, *DLO2* and *DMR6*) encoding proteins belonging to 2OG and Fe(II) dependent oxygenase superfamily. In a recent study in Arabidopsis, it was demonstrated that loss of function of PRT1, involved in N-degron pathways of ubiquitin-mediated proteolysis, improves the plant immune system. DMR6 and DLO1 proteins were accumulated in the former study indicating their importance in regulating the basal defense system^77^.

Several members of the NAC TF family such as *ATAF2*, *NAC102* and *NAC032* have been shown to be regulated by NO^78,79^. In accordance with the former studies, two transcripts (Solyc04g009440 and Solyc11g017470) annotated as *ATAF2/NAC081*, showed up-regulation in the current study in response to hypoxia and SNP-treatment (Fig. 6). NAC TFs are involved in plant development and response to different abiotic stresses^80^. The expression of *ATAF2/NAC081* TF in maize has been shown to be positively regulated by ZmPTF1. ZmPTF1 is a member of the basic helix-loop-helix (bHLH) family involved in phosphate starvation and drought tolerance as well as root development in maize. ZmPTF1 binds to the G-box element within the promoter of several TFs such as *ATAF2/NAC081* and *NAC30*^81^. Up-regulation of the above-mentioned genes has been reported to be involved in root development and stress response^81^. These data are in line with the up-regulation of *ATAF2/NAC081* TF in our study indicating that it might be involved in root hypoxia response. However, whether NO is involved in *ATAF2/NAC081* regulation requires further investigation.

*NAC083*, also known as *VND-INTERACTING2 (VNI2)*, showed down-regulation in response to hypoxia and SNP-treatment in the current study. NAC083/VNI2 is an ABA responsive TF which has been shown to be involved in plant stress response. High salinity has been shown to increase the expression of *NAC083/VNI2* in an ABA-dependent manner. Moreover, NAC083 negatively regulates stress-induced leaf senescence through regulation of *COLD REGULATED* (*COR*) and *RESPONSIVE TO DEHYDRATION* (*RD*) genes^82,83^. Two abiotic stress marker genes,* COR15A/B* and *RD29A/B* have been shown to be regulated by direct NAC083/VNI2 binding to their promoter. In the current study, only *RD22* showed down-regulation which is in line with *NAC083/VNI2* down-regulation^83^. Our data suggest that the down-regulation of *NAC083/VNI2* under hypoxia might be NO-dependent. However, how hypoxia and NO results in repression of *NAC083/VNI2* in the root, requires more investigations and might provide more insight into the role of *NAC083/VNI2* in the regulation of hypoxia response.

*WRKY7* showed up-regulation in the current study (Fig. 6). Up-regulation of WRKY encoding transcription factors, such as *WRKY22*, in response to submergence, has been reported in Arabidopsis, resulting in induction of immunity related marker genes^84^. *wrky22* mutant showed the lower expression level of defense related genes after submergence^84^. These data indicate that there is a link between submergence induced hypoxia and defense in plants. However, the underlying signaling network, particularly the role of NO, is not yet unraveled.

*REIL1*, a member of the C2H2 zinc finger family, showed the highest up-regulation in our study in response to hypoxia (17.3-fold) and SNP-treatment (12.1-fold) (Fig. 6). REIL1 and REIL2 have been shown to be involved in *A. thaliana* leaf growth in the cold but not in normal temperature^85^. REIL1 provides an interesting candidate for further investigating its role in response to NO and hypoxia tolerance.

### Genes related with ROS metabolism were regulated in response to hypoxia and SNP-treatment

Genes encoding different categories of ROS associated proteins such as peroxidases, oxidases, nitrilases, glutathione *S*-transferases as well as cytochrome P450 showed up- and down-regulation in the current study (Fig. 8).

Accumulation of ROS and reactive nitrogen species (RNS) is associated with low oxygen stress^53,86,87^. NO and its derivatives such as peroxynitrite (OONO^-^), dinitrogen trioxide (N_2_O_3_), and nitrous acid (HNO_2_) have been reported to be involved in the modification of cellular redox statues^88,89^.

Among the genes, regulated under hypoxia and SNP-treatment, two Cytochrome P450 encoding genes (*CYP72A14* and *CYP707A3*), showed the highest up-regulation, > 30 and > 6-fold, respectively, in response to hypoxia (Fig. 8). Cytochrome P450 (CYP450) can convert toxic metabolites (e.g. superoxide anion, hydrogen peroxide and hydroxyl radical) to H_2_O_2_ to prevent harmful effects on the cell. Therefore, CYP450s are considered as markers for oxidative stress.

High level of ROS has to be scavenged by the cellular antioxidant system consisting of enzymatic antioxidants (e.g. superoxide dismutase (SOD), catalase (CAT) and glutathione peroxidases (GPXs), thioredoxin (Trx)) and non-enzymatic antioxidants (e.g. ascorbic acid, glutathione (GSH), carotenoids)^90^. Accordantly, up-regulation of genes encoding different classes of ROS scavenging enzymes were observed. Among six regulated GST encoding genes in our study, *GSTU8* showed the highest up-regulation (ca. 6-fold) under hypoxia (Fig. 8). It has been shown that Arabidopsis *gstu8* mutant line does not demonstrate any phenotypic changes nor modifications in the glutathione profile. However, it became evident that interaction between different dehydroascorbate reductases (DHARs) mediates the link between ascorbate and glutathione pools to ensure glutathione associated signaling under excessive H_2_O_2_^91^.

*MT2B*, encoding metallothionein-like protein 2B, a ROS scavenger, showed down-regulation in our study (Fig. 12). It has been shown that *MT2B* down-regulation is associated with ROS accumulation and subsequent aerenchyma formation in rice^64^. This indicates that MT2B might be involved in hypoxia tolerance in tomato root. However, the role of NO during this process has not yet been addressed.

### Hypoxia and SNP-treatment led to the down-regulation of AQP encoding genes

Members of two families of aquaporin (AQP) encoding genes (PIPs: 12 genes and TIPs: 7 genes) were down-regulated in our study in response to hypoxia and SNP-treatment (Fig. 9).

AQPs are involved in the transport of water and other small molecules such as ammonia, boron, CO_2_, H_2_O_2_ and urea across membranes^92^. Moreover, AQPs have been shown to be involved in plant biotic and abiotic stress response^93^. Expression reduction of NtAQP1, a member of the PIP1 family, in tobacco, led to a decrease in hydraulic conductivity of the root and eventually reduced drought stress resistance^94^.

In accordance with our results, microarray analysis showed that O_2_ deficiency resulted in the down-regulation of AQPs in *Arabidopsis* (Liu et al., 2005) and Avocado^95^. It has been shown that beside modifications in cytosolic Ca^2+^ and H_2_O_2_ level, low cytosolic pH during anoxia results in inhibition of hydraulic conductivity through a mechanism of pH dependent AQP gating^96^. During flooding, AQPs remain phosphorylated but closed due to the protonation of His193 in PIP2;1 of spinach plants^97^.

Tobacco PIP1;3 has been shown to be potentially involved in the O_2_ transmembrane transport^98^. Hypoxia stress in hydroponically grown tobacco, resulted in the up-regulation of *PIP1*;*3* in the whole root^98^ and its down-regulation in lateral (LR) but not adventitious (AR) roots. The latter study demonstrated that beside *PIP1*;*3*, *PIP1*;*1* was also down-regulated after 2 days of hypoxia treatment. Moreover, down-regulation of other AQP encoding genes such as *PIP1*;*2*, *PIP1*;*4*, *PIP2*;*1* in LR, was observed after one week of hypoxia treatment^99^. The authors did not observe any difference in hydraulic conductance (K_r_) between hypoxic and aerated plants, indicating the efficiency of AR in root water transport in tobacco. The down-regulation of PIP genes in our study is in accordance with AR response in tobacco. However, our study was focused on the whole root (Fig. 9). It has been reported that hypoxia is associated with a lower Kr in some species but an unchanged Kr in the others^100,101^. Tomato plants grown in soil, showed an early negative root hydraulic conductivity in response to flooding without affecting stomata closure^102^. It remains to determine whether expression changes in PIPs encoding genes is correlated with the functionality of PIPs and Kr in tomato root in response to hypoxia.

TIPs are involved in water transport between the vacuole and cytoplasm and therefore play a role in the regulation of cellular turgor pressure in plants^93,103^. One of the down-regulated TIP encoding genes in our study was TIP2;2 (Fig. 9). Overexpression of TIP2;2, in tomato, improved drought stress tolerance of transgenic plants through regulation of transpiration rate. This indicates that the water permeability rate across tonoplast is involved in drought stress tolerance^104^. Our data suggest that studying expression changes as well as activity of TIP2;2 during flooding induced hypoxia gives more insight into the importance of vacuole-cytoplasm water relation in flooding stress tolerance. It is noteworthy that protein storage vacuoles (PSVs) in stem cell niche and lytic vacuoles (LVs) in mature cell, contain distinct TIPs proteins in their membran^105^. Further studies are required to unravel the response of cell type and vacuole specific TIPs to nitric oxide and flooding.

NO transport through AQPs, as well as its potential role in expression regulation of AQPs, is not yet clear. In a human cell line expressing AQP1, NO permeability across the cell membrane was correlated with water permeability^106^. In the latter study, NO transport was significantly reduced after the addition of HgCl_2_, an aquaporin inhibitor. The authors concluded that NO transport by AQP1 controls intracellular NO levels and its consequences^106^. However, whether NO transport and controlling its cellular level during hypoxia can be executed via aquaporins in plant cells, needs further investigations.

This result indicates that little is known about the role of AQPs in response to hypoxia and the role of NO in this process is still unknown. Gene regulation does not necessarily correlate with protein amount and function. Transcriptional, post-transcriptional and posttranslational regulation can eventually affect the amount and the activity of AQPs. Moreover, the clear link between NO and AQPs under hypoxic conditions is not yet fully known. Our result indicates the necessity of future investigations to unravel the functional significance of AQPs and NO in root hypoxia tolerance.

### Stress associated genes showed expression changes under hypoxia and SNP-treatment

Among stress related genes, *SRO5 (SIMILAR TO RCD ONE 5*) showed the highest up-regulation level in response to hypoxia (22.2-fold) in our study (Fig. 10). *SRO5* has already been reported to be a common hypoxia-responsive gene, particularly in the root, throughout the plant kingdom^65,107,108^. The role of SRO5 in salt stress has been addressed in Arabidopsis. *SRO5* overexpression led to a reduction in root H_2_O_2_ content in response to salt stress, compared to WT and *sro5* plants^109^. Our data suggest that SRO5 might play a role in alleviating H_2_O_2_ level during hypoxia stress. Further investigations are required to unravel the link between hypoxia-induced NO and SRO5 in tomato root.

Among the stress related genes, *Heat shock protein 23.6* (*HSP23.6*) showed the highest up-regulation under hypoxia. SNP application in *Arabidopsis* has shown that different members of the heat shock TF family are responsive to NO^110^. Moreover, HSPs and HSFs are also responsive to anoxia^111,112^. These data indicate that hypoxia-induced NO production might be involved in the regulation of HSPs. The possible cross talk between hypoxia and NO-mediated thermo-tolerance and HSPs chaperon function remains to be determined.

### Comparison to former hypoxia and NO studies

Common regulated genes in response to hypoxia and SNP-treatment (FDR < 0.05) in our study were compared with the data obtained from a well-designed former study on Arabidopsis seedlings^48^. In the former study, 357 genes showed regulation changes in response to hypoxia in WT. Out of the above-mentioned genes, only four genes were shared with our study (Supplementary Table S3). This might be related to the fact that in our study only roots were exposed to hypoxia and SNP in a hydroponic system but in the other study, the whole seedlings were under submergence. Moreover, the plant age, 5 weeks old tomato vs. Arabidopsis seedlings, might partially explain the low overlap of the regulated genes between both studies. Moreover, regulated genes in our study were compared with the genes regulated in a triple NO mutant (*nia1nia2noa1-2*)under normoxia relative to WT^48^. 70 out of 251 (28%) genes in the current study were also regulated in the triple NO mutant under normoxia. Concomitantly, 51 out of 70 genes (73%) were regulated similarly in both studies (Supplementary Table S4).

The Arabidopsis N-degron pathway mutants (*prt6 and ate1ate2*) have been reported to exhibit constitutive expression of several core hypoxia genes (e.g. *ADH1*, *SUS4* and *PDC1*) under normoxia leading to better resistance against hypoxia^48^. To identify the possible targets of the N-degron pathway in our study, regulated tomato genes were compared with the above-mentioned mutants in Arabidopsis. The result showed 13 common genes between tomato and Arabidopsis *prt6* mutant*.* 10 genes, among them *SUS4*, were up-regulated (Supplementary Table S5). Comparison of our results with Arabidopsis *ate1ate2* mutant revealed that 12 genes were common between two data sets, with 8 genes showing similar regulation changes (Supplementary Table S6).

**Figure 13:**
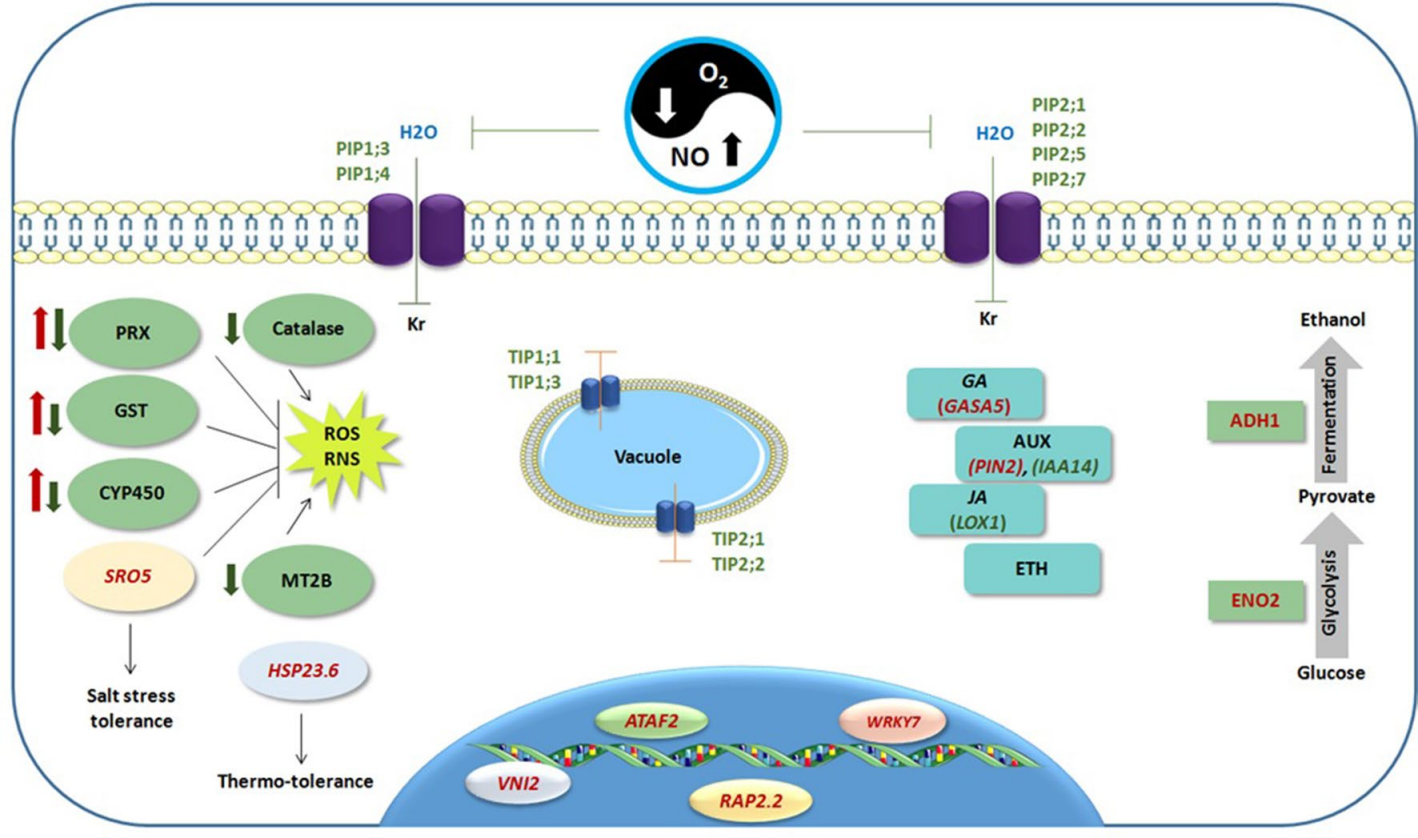
A schematic model of the molecular response of tomato root to the yin and yang of oxygen and nitric oxide. Depicted are the genes belonging to different biological categories with identified or proposed roles in response to hypoxia and/or NO. Red and green font colors or arrows represent up- and down-regulation, respectively. *ADH1*, *ALCOHOL DEHYDROGENASE 1*; *ENO2*, *ENOLASE 2*;* CA1*, *CARBONIC ANHYDRASE 1*; *CA2*, *CARBONIC ANHYDRASE 2*; Kr: hydraulic conductivity, *TIP*, *tonoplast intrinsic proteins*; *PIP*,* plasma membrane intrinsic protein*s; PRX, peroxidase; GST, glutathione S-transferase; *MT2B*, *METALLOTHIONEIN 2B*;*CYP450*,*cytochrome P-450*; *SRO5*,* SIMILAR TO RCD ONE 5*; HSP23.6, *HEAT SHOCK PROTEIN 23.6*; *ATAF2 (NAC081)*, *ARABIDOPSIS NAC DOMAIN CONTAINING PROTEIN 81*; *WRKY7*, *WRKY DNA-BINDING PROTEIN 7*; *VNI*2 (NAC083), *VND-INTERACTING 2*;* RAP2.2*,* RELATED TO AP2 2*; *GASA5*, *GAST1 PROTEIN HOMOLOG 5*; *PIN2*, *PIN-FORMED 2*; *IAA14*,* INDOLE-3-ACETIC ACID INDUCIBLE 14*;* LOX1*, *LIPOXYGENASE 1*;* GA*,* gibberellic acid*;* AUX*,* auxin*;* JA*,* jasmonic acid*;* ETH*,* ethylene*.

In summary, our data suggest an overlap in gene expression response to long-term hypoxia and SNP-treatment. The concertedly regulated genes belong to different biological categories such as phytohormone signalling and transcription factor related genes as well as genes which their encoded proteins are involved in various metabolic pathways such as redox regulation, transport across membrane, glycolysis and fermentation. It can be proposed that the identified genes in our study could be considered as targets of hypoxia-induced NO and requires more investigation to unravel their role in the anatomic and metabolic adjustment of long-term hypoxia response and tolerance in tomato root. Beside TFs and phytohormones, the emphasis of the future studies needs to be placed on investigating the function of redox regulated proteins and their interaction with NO under hypoxia. These findings are essential to understand the cellular control of stress induced ROS/RNS as signal molecules as well as harmful radicals for the cell during hypoxia. A schematic model is illustrated to summarize the genes addressed in the discussion section (Fig. 13). It is noteworthy that this study was conducted on the RNA extracted from the whole root. Future investigations on phenotyping with cell type map and Single-cell RNA-seq approach can provide a more precise view on the cell type specific response and gene expression changes in response to nitric oxide and flooding stress.

## Methods

### Plant material and growth conditions

Plant age and growth condition was the same for both hypoxia and SNP studies. The hypoxia treatment has been previously described in details^53^. Tomato plants (*Solanum lycopersicum* L. cv. Moneymaker) were grown on sand in the greenhouse (500 μmol photons/m^2^/s and 25 °C under a 14/10‐h light/dark) for three weeks. During this time, a modified Hoagland nutrient solution containing 5 mM nitrate (NO_3_^-^), as described previously^113^, was used for treatment. Three-week-old plants were transferred to hydroponic plastic boxes containing 6 L of nutrient solution (pH 5.5). Roots were submerged in the nutrient solution and aerated by mild bubbling using aquarist air pumps (Hailea ACO-9620, Raoping, Guangdong, China) and air outlets (Tetratec, Osnabrück, Germany). The hydroponic boxes were covered with dark covers, so that the roots were kept in dark. Despite dark, the low pH of the nutrient solution around the root will facilitate the release of NO from SNP^114^. The hypoxia treatment was conducted on five weeks old plant roots using N_2_ gas (≥ 99.99 Vol. %) (Air Liquide, Germany) for 48 h^53^. For SNP-treatment, the root of five weeks old plants was treated with final concentration of 500 μM SNP. The roots were harvested 48 h after the initiation of SNP-treatment in the solution for this study. Hypoxia was induced via root exposure to N_2_ gas^53^. Both treatments were started at 8:00 a.m. and continued 48 h until the harvest time at 8:00 a.m.

A Konica Minolta SPAD-502 chlorophyll meter was used to measure the relative chlorophyll levels of leaf #3 (the third leaf above cotyledones)^53^.

### Read mapping and identification of differentially expressed genes

All the processes involved in data mapping and analysis has been described in a former study^53^. Adaptor clipped reads obtained from the NextSeq500 Illumina platform (LGC Biosearch Technologies, Berlin, Germany) were used for the following processes. After omitting short fragments and low quality reads, rRNA sequences were filtered. Remaining sequences were mapped to tomato reference genome (ITAG 2.4) (The Tomato Genome Consortium, 2012) using CLC Genomics Workbench (Qiagen, V. 7.5.5). Adaptor clipped reads obtained from the NextSeq500 Illumina platform (LGC Biosearch Technologies, Berlin, Germany. Sequencing data are deposited in the Sequence Read Archive (SRA) database (bioproject accession PRJNA553994) at the National Centre for Biotechnology Information (NCBI). The bioproject’s metadata are available at https://dataview.ncbi.nlm.nih.gov/object/PRJNA553994 reviewer = 684sto9a948tin240f0tlt1o1h.

The TMM (trimmed means of M values)^115^ and the edgeR algorithm^116^ were used for normalization and estimation of P-values, respectively. The algorithm edgeR was used for log fold change values. The P-values were adjusted for multiple testing^117^. All calculations were performed with the CLC Genomics Workbench software (Qiagen, V. 7.5.5). Differentially expressed genes (DEGs) (P_adj_ < 0.05) (Supplementary Table S1) were selected for subsequent analysis. The FDR threshold was used for the P-value in multiple tests (P_adj_). GO term enrichment analysis (P_adj_ < 0.05), was performed using the Panther database^118,119^. For biological pathway analysis of differentially regulated genes, MapMan categories based on ITAG 2.3 annotations^54^ were used. Heat maps of differentially regulated genes were created using MultiExperiment Viewer (MeV 4.9.0)^120^.

### RNA isolation and cDNA synthesis

The whole root was snap-frozen and grounded in liquid N_2_. 250 mg of the homogenized grounded powder was used for total RNA extraction following phenol–chloroform extraction method^121^. The integrity of RNA was checked on 1.2% agarose gel. RNA concentration was quantified photometrically using NanoDrop (ND-1000, Thermo Scientific, Wilmington, DE, USA).

cDNA synthesis was performed with two μg DNase I-digested total RNA as template and using oligo-(dT)_18_ and RevertAid H Minus First Strand kit (Thermo Scientific, Waltham, USA).

### qPCR primer design and assay

Primers for quantitative real-time PCR (qPCR) were calculated by QuantPrime^122^ (Supplementary Table S2). qPCR reactions were performed in total volume of 5 μl including 2.5 μl Power SYBR Green Master Mix (Thermo Fisher Scientific), 0.5 μM forward and reverse primers and 0.5 μl cDNA. *ACTIN* was used as reference gene^123^. The thermal profile used for all qPCRs was: 2 min 50 °C > 10 min 95 °C > (15 s 95 °C > 1 min 60 °C)_40x_. Data were analyzed by the 2^−ΔΔCt^ method^124^.

## Supplementary information

Supplementary Tables

## Data Availability

All materials and data sets represented in the current study are available in the main text or the supplementary materials. RNA-Seq. data are deposited to the Sequence Read Archive (SRA) database (bioproject accession PRJNA553994) at the National Center for Biotechnology Information (NCBI). The bioproject’s metadata are available at https://dataview.ncbi.nlm.nih.gov/object/PRJNA553994 reviewer = 684sto9a948tin240f0tlt1o1h.
